# Novel myristoylation of the sperm-specific hexokinase 1 isoform regulates its atypical localization

**DOI:** 10.1242/bio.012831

**Published:** 2015-11-18

**Authors:** Sujeet Kumar, Sreejit Parameswaran, Rajendra K. Sharma

**Affiliations:** Department of Pathology and Laboratory Medicine, Cancer Cluster, College of Medicine, University of Saskatchewan, 107 Wiggins Road, Saskatoon, Saskatchewan S7N 5E5, Canada

**Keywords:** Hexokinase, Myristoylation, Protein acylation, Atypical localization, Alternative splicing

## Abstract

The hexokinase 1 variant in mammalian spermatozoa (HK1S) has a unique N-terminus and this isoform atypically localizes to the plasma membrane. However, the mechanism of this process currently remains ambiguous. In this report, we show that fatty acylation underlies the specific sorting of HK1S. Employing chimeric reporter constructs, we first established that compartmentalization of HK1S does not function exclusively in sperm cells and that this feature is swappable to somatic HEK293 cells. Although the N-terminus lacks the classical consensus signature for myristoylation and the sequence-based predictions fail to predict myristoylation of HK1S, complementary experimental approaches confirmed that HK1S is myristoylated. Using live-cell confocal microscopy, we show that the mutation of a single amino acid, the myristoyl recipient Gly^2^, impedes the prominent feature of plasma membrane association and relocates the enzyme to the cytosol but not the nucleus. Additionally, substitutions of the putatively palmitoylated Cys^5^ is also reflected in a similar loss of compartmentalization of the protein. Taken together, our findings conclusively demonstrate that the N-terminal ‘MGQICQ’ motif in the unique GCS domain of HK1S acquires hydrophobicity by dual lipidic modifications, N-myristoylation and palmitoylation, to serve the requirements for membranous associations and thus its compartmentalization.

## Abstract (precis)

**Summary:** Dual acylation (i.e. myristoylation and palmitoylation) regulates the atypical localization of sperm-specific hexokinase 1 isoform and thus is essential for ‘rewiring’ of glycolytic network.

## INTRODUCTION

The diversified cellular requirements within a constantly changing environment have made cells to evolve mechanisms that facilitate their capability to flexibly regulate signaling and metabolic functions. Therefore, even for extensively characterized proteins, deciphering protein functions is highly enigmatic when precisely delineating how they perform their roles in a specific cellular context (Henderson and Martin, 2014). Numerous enzymes of the glycolytic pathway reflect such dynamic behavior in which the prevailing view of the individual glycolytic enzymes as proteins with well-designated catalytic functions and structural properties has been revised (Jeffery, 2009; Jung et al., 2014b; Menard et al., 2014). Multiple glycolytic enzymes have been more recently classified as proteins with non-canonical roles (Dai et al., 2008; Jung et al., 2014a; Rawat et al., 2012; Subramanian and Miller, 2000). This is conservative and efficient on the part of the organism, but it requires locational re-organization of the proteins to keep functions tightly regulated.

Hexokinases (ATP:D-hexose 6-phosphotransferase; EC2.7.1.1; HKs) carry out the first committed step in glucose metabolism by catalyzing the phosphorylation of glucose to yield glucose-6-phosphate (G6P) (Wilson, 2003). Among the major hexokinase isoforms: HK1, HK2, HK3, and HK4 (also known as glucokinase), HK1 is the most ubiquitous isoform that associates with mitochondria via its porin binding domain (PBD) in the N-terminal segment of the protein (Sun et al., 2008; Wilson, 2003). It is known that male germ cells have an unique architecture for glycolysis in which the glycolytic enzymes are clustered in the fibrous sheath (FS) region of sperm cells (Krisfalusi et al., 2006; Nakamura et al., 2010). The male germ cells (spermatozoa) have a conserved structural organization among mammals which is specialized both morphologically and biochemically to deliver the male genome to the egg. The sperm flagellum contains the machinery required for motility as demonstrated by the targeted gene disruption of enzymes; that confirmed ATP generated via glycolysis is essential for sperm motility and male fertility (Danshina et al., 2010; Miki et al., 2004; Nakamura et al., 2013; Narisawa et al., 2002; Odet et al., 2013).

The tethering of multiple glycolytic enzymes to the FS of the mammalian sperm is highly resistant to extraction; however, HK1 as an exception is readily solubilized by detergents and is not identified in the isolated fibrous sheath preparation (Kim et al., 2007; Nakamura et al., 2010). Three germ cell-specific HK1 transcripts have been identified in mice and humans, but only one is expressed at the protein level (Mori et al., 1996; Travis et al., 1998). The spermatogenic cell type HK1 (HK1S) possesses a replacement of the PBD of the somatic cell type (normal) HK1 isoform (HK1N) by an unique germ cell-specific (GCS) domain generated by alternative splicing (Andreoni et al., 2000; Nakamura et al., 2008).

Previous reports indicate the presence of HK1S on the sperm surface, suggesting its probable role in sperm and egg zona pellucida interaction (Travis et al., 1998). To sustain the flux of G6P into the tethered arrangement of glycolytic enzymes in FS, HK1S localization is critical for promoting the biological roles conferred by its specific compartmentalization. Previous reports demonstrate that HK1S is uniquely targeted to the FS in a manner that is sensitive to treatment with Triton X-100, therefore suggesting a hydrophobic association. However, other groups have noted that the GCS domain is responsible for the compartmentalized location (Mori et al., 1998; Travis et al., 1998). The peripheral localization of HK1S is also suggested to be dependent upon its association with another spermatogenic cell-specific variant isozyme of the glycolytic enzyme muscle-type phosphofructokinase (PFMKS) (Nakamura et al., 2010). Despite the established roles of HK1S in sperm functions, the mechanism mediating its atypical localizations has remained elusive. In the current report, we show that the differential targeting of the HK1S to the plasma membrane periphery (PMP) of the cell is an integral feature of the molecule. The GCS region of HK1S lacks the consensus sequence for myristoylation, but experimental validations show that the penultimate glycine in the unique N-terminal region serves as an acceptor for the myristoyl group. HK1S, thus acquires hydrophobicity through the attachment of lipidic moiety in its unique N-terminal region. Our results demonstrate that the dual acylation sites in the N-terminal motif ‘MGQICQ’ within the GCS domain act in concert as the key spatial determinants for the proper plasma membrane localization. HK1S atypical localization is thus a reflection of the interplay between the alternative splicing that gives rise to a unique N-terminus and the lipidic modification conferred on to this region.

## RESULTS

### The plasma membrane localization of sperm-specific HK1 is an integral feature of the protein

**Fig. 1.**
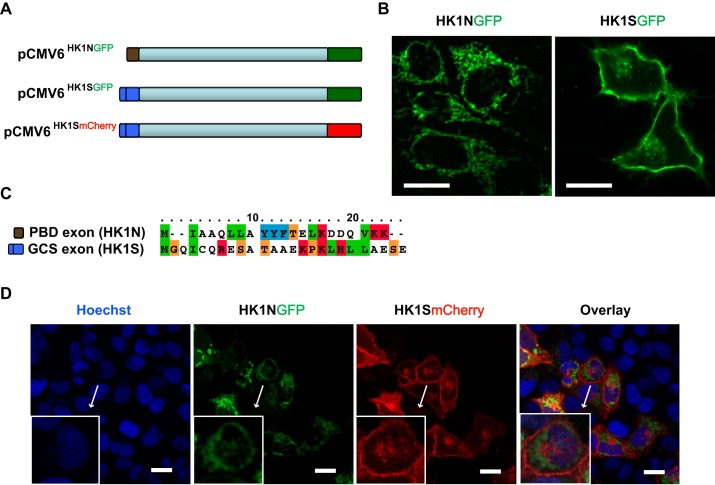
**Sorting specificity of HK1S is maintained in cells of somatic origin.** (A) Schematic representation of the chimeric reporter system for the human HK1 isoforms depicting tandem fusion with the fluorescent protein markers GFP (green) and mCherry (red). (B) Representative cells exhibiting distinct compartmentalization of HK1N and HK1S in HEK293 cells transiently transfected with pCMV6^HK1NGFP^ and pCMV6^HK1SGFP^. HK1N^GFP^ localizes to the distinct membranous regions within cytoplasm (left panel), whereas the HK1S^GFP^ localization is predominantly to PMP regions of the cell (right panel). Images were obtained 24 h post-transfection. (C) Comparison of the amino acid sequences of PBD and GCS region of human HK1N and HK1S isoforms reflecting the significant dissimilarity. The coloring of the residue types corresponds to the default scheme in CLUSTALX. (D) Confirmation of the distinct compartmentalization of pCMV6^HK1NGFP^ and pCMV6^HK1SmCherry^ as indicated by the co-localization. Twenty-four hours post-transfection, cells were treated with permeable nuclear dye (Hoechst dye 33258; Sigma), and imaged by live cell confocal microscopy to detect nuclei (blue), HK1N^GFP^ (green) and HK1S^mCherry^ (red). The merged images are presented in the far-right column. The inset shows the distinct localization of HK1N^GFP^ and HK1S^mCherry^ within a single cell. Scale bars=20 μm.

In mammalian sperm cells, the localization of HK1S to the FS in the vicinity of PMP is mediated by tethering of its unique GCS domain to PFKMS (Nakamura et al., 2010). The GCS domain in isolation does not confer the sorting specificity when expressed in heterologous NIH3T3 cells (Nakamura et al., 2003). However, the sorting specificity is maintained when the full-length protein is expressed in M+R42 cells, albeit lacking the HK (Travis et al., 1999). In light of this anomaly, we therefore first sought to establish if the compartmentalization of HK1S to the PMP regions of sperm cells is a germ-cell specific hallmark. We hypothesized that if the peripheral localization is an integral feature of the HK1S molecule, it should be emulated in the cells of somatic origin. We employed the widely established representative system of mammalian cell lines, HEK293 cells, as a model system towards the understanding of the atypical localizations of HK1S. We engineered a C-terminal green fluorescent protein (GFP)-tagged version of HK1S (HK1S^GFP^) and HK1N (HK1N^GFP^), and transfected the expression plasmids into HEK293 cells (Fig. 1A). The GFP signals emanating from the chimeric fusion constructs were analyzed by live-cell confocal microscopy to observe the localizations of these proteins. The GFP signal from HK1N^GFP^ (Fig. 1B, left panel) is in a pattern reminiscent of its mitochondrial localization, which has been previously reported (John et al., 2011; Sun et al., 2008). However, we discovered that in the HEK293 cells, HK1S^GFP^ localize to the PMP regions of the cell (Fig. 1B, right panel) in contrast to the localization of HK1N^GFP^ in defined regions within the cytoplasm (Fig. 1B, left panel). The sequence difference clustered within the PBD and GCS of HK1N and HK1S, respectively, is highlighted in Fig. 1C. The HEK293 cells are of embryonic kidney tissue origin and lack the PFKMS components of the sperm cell-specific glycolytic enzymes (Nakamura et al., 2010). Our findings thus validate that the specific compartmentalization of HK1S is independent of its association with PFKMS. The plasmid pCMV6^GFP^ was used in simultaneous transfections to account for the comparative localization of the isolated GFP (Fig. S1, top left panel) that displayed its characteristic diffuse cytosolic and nuclear distribution (Sun et al., 2008).

To verify that the localization of HK1S does not overlap with the mitochondrial localization of HK1N, we employed a co-transfection assay and observed the distinct compartmentalization of the somatic and sperm specific HK1 isoforms, HK1N^GFP^ and HK1S^mCherry^, respectively (Fig. 1D). The red variant of GFP (mCherry) in isolation retains the diffuse cytosolic and nuclear distribution (Fig. S1, top right panel) pattern of GFP (Fig. S1, top left panel). To confirm the sorting specificity, we employed HK1S^mCherry^ and observed its localization contrasted with the dye MitoTracker Green. As expected, the HK1S^mCherry^ signals (red) remain peripheral and having no overlap with the mitochondrial staining (green), thus confirming that HK1S does not localize to the mitochondria in HEK293 cells (Fig. S1, bottom panel). The observations suggest that the atypical localization of HK1S, to the FS in the vicinity of the plasma membrane in male germ cells, is an integral feature of the molecule and is therefore able to resonate in HEK293 cells by its localizations to the PMP of the cell (Fig. 1B,D).

### Sequence-specific features in the divergent N-terminus region of HK1N and HK1S

It is established that HK1S arises from the HK1N gene by an alternative splicing, utilizing a germ cell specific promoter and the differences are uniquely clustered in the N-terminal region (Andreoni et al., 2000; Nakamura et al., 2008). To analyze the nature of amino-acid changes, we identified the human HK1N and HK1S variants from the CCDS database (CCDS ID: CCDS7292.1 and CCDS7289.1 respectively) (Pruitt et al., 2009). The CCDS sequence feature of the divergent region reflects that the PBD of HK1N is contained within the first exon, which is replaced by the GCS region encompassed over first two exons in the HK1S. The shuffling of the PBD-containing exon of HK1N by the GCS region in HK1S completely diversifies the nature of amino acids in this region and extends the sequence length by four amino acids (Fig. 1C). The single-residue determinants essential for the interaction of HK1N with the mitochondrion are well-established. In addition to the hydrophobic nature of the PBD, residues Ala^4^, Gln^5^ and Ala^8^ are highly critical to mitochondrial binding as individual mutations at these sites cause a 10-fold decrease in HK1N binding to mitochondria (Mehyar et al., 2011). However, the replacement of PBD with GCS in HK1S results in the loss of these critical features (Fig. 1C). The membranous association of HK1S in FS has been reported to be sensitive to Triton X-100, but not to NaCl or Na_2_CO_3_ (Travis et al., 1998). This indicates that the HK1S compartmentalization is mediated by hydrophobic interactions. However, the mechanism behind the targeting of HK1S to peripheral membranous regions by the hydrophobic associations remains elusive. We noticed that HK1S contains a glycine residue that is present downstream of the initiator methionine (Fig. 1C). This raises the alternate possibility that the penultimate glycine could serve as an acceptor for C14:0 fatty acid (i.e. myristic acid) (Maurer-Stroh et al., 2002b; Resh, 2006). The protein modification with the 14-carbon long hydrophobic moiety could thus possibly promote protein–membrane interaction (Resh, 1999). We therefore performed myristoylation prediction analysis on the GCS domain to discern whether the sequence qualified as a motif for myristoylation by NMT. We used *Myristoylator* that discriminates myristoylated and non-myristoylated proteins by an ensemble of neural networks and the *Myr predictor* that utilizes a scoring system based on sensitive profile extraction, physical property requirements and compensatory effects to be recognized by NMT (Bologna et al., 2004; Maurer-Stroh et al., 2002a). Contrary to our surmise, both evaluations failed to predict the N-terminal glycine in GCS as a possible N-terminal myristoylation site in the HK1S (Table 1).

**Table 1.**
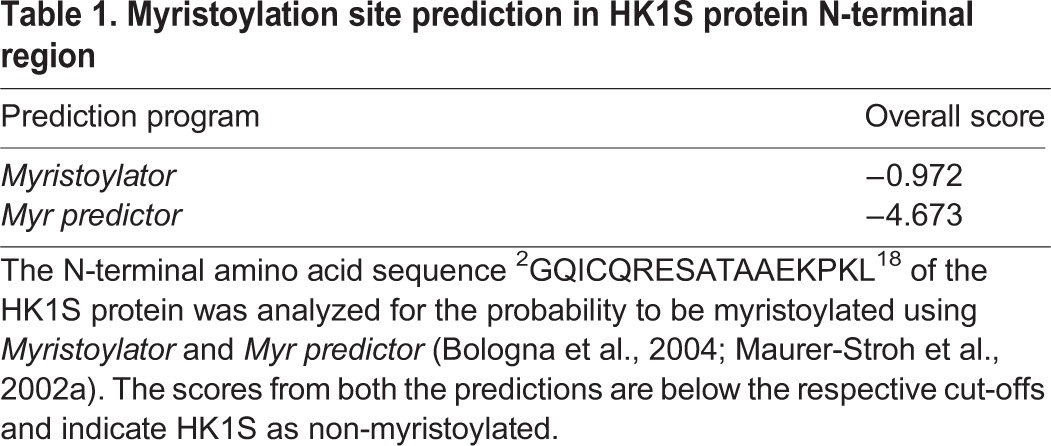
**Myristoylation site prediction in HK1S protein N-terminal region**

### HK1S is myristoylated on *Gly^2^* in its unique N-terminal region

**Fig. 2.**
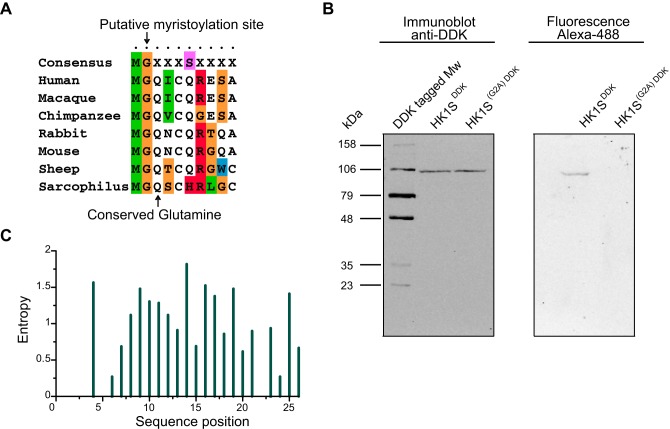
**N-terminal Gly^2^ residue in HK1S is myristoylated.** (A) Comparison of the consensus sequence for protein *N*-myristoylation with orthologous HK1S. Alignment of the N-terminus amino acids 1-10 of HK1S from various mammalian species demonstrates the absolute conservation of the terminal ‘MG’ motif in this region and the Gln^3^ critical for myristoylation in absence of Ser^6^/Thr^6^. The Ser^6^ on the consensus motif is highlighted in pink. (B) Co-translational myristoylation of HK1S on Gly^2^ in a rabbit reticulocyte *in vitro* translation assay. HK1S^DDK^ and HK1S^(G2A)DDK^ were subjected to coupled TNT assay in the presence of *Az*Myr. Labeled proteins were affinity enriched on anti-DDK beads and analyzed by chemical proteomics strategy. Immunoblotting by DDK-tag specific monoclonal antibody reflects full-length synthesis for both the proteins HK1S^DDK^ and HK1S^(G2A)DDK^ (left panel). Incorporation of *Az*Myr into HK1S^DDK^ but not HK1S^(G2A)DDK^ detected by ‘click-chemistry’ (right panel) indicates the specific myristate labeling of HK1S on Gly^2^. Positions of indicated molecular size markers are in kilodaltons (kDa). (C) Entropic analysis of the sequence divergence in the GCS domain. The sequence position corresponds to the alignment in Fig. S3A. The height of the bars indicates relative variation at the particular sequence positions (0, least variable; 2.996, most variable).

The sequence comparison of the N-terminal region of HK1S among the corresponding proteins from other mammals reflects that the terminal Met-Gly (MG) motif is absolutely conserved (Fig. 2A). Proteins destined to be N-myristoylated are modified on the α-amino group of Gly^2^ after the co-translational removal of the initiator methionine by methionine-aminopeptidase and myristic acid is linked to the liberated glycine via an amide bond in the NMT catalyzed reaction (Resh, 2006). The usual consensus signature for myristoylation is defined as MGXXXS/T (Resh, 1999), but none of the homologous proteins of HK1S embody Ser^6^/Thr^6^ conferring to the consensus N-myristoylation motif (Fig. 2A). However, Ser^6^/Thr^6^ is neither sufficient nor critical for protein *N*-myristoylation (Maurer-Stroh et al., 2002b). The N-terminal ‘MG’ motif is the absolute requirement while the other features could be varying (Maurer-Stroh et al., 2002b, 2004). Along with the conserved Gly^2^, another prominent observation is the absolute conservation of Gln^3^ (Fig. 2A), a feature essential for N-myristoylation of proteins lacking Ser^6^/Thr^6^ (Utsumi et al., 2001). The generality of these observations provided compelling evidence and we therefore sought to challenge the *N*-myristoylation status of HK1S utilizing two complementary experimental approaches. We first validated that the N-terminus of HK1S is a candidate for protein myristoylation. The N-terminal octapeptide residues lacking the initiator methionine and encompassing GXXXXXXX residues serve as efficient substrate in the *in vitro* myristoylation reactions (Traverso et al., 2013). Utilizing the synthetic HK1S N-terminal octapeptide, ^2^GQICQRES^9^-CONH_2_, we performed an *in vitro* myristoylation assay, followed by MS analysis, as previously described (Kumar and Sharma, 2014). The control reactions (i.e. lacking the donor myristoyl-CoA) reflects two m/z peaks of 919.43 and 1835.85 that harmonize to the monoisotopic masses of the ^2^GQICQRES^9^-CONH_2_ peptide and the corresponding Cys^5^-Cys^5^ dimer, respectively (Fig. S2A**)**. The proteomic identification of the complete reaction reflects the presence of three m/z peaks of 919.43, 1129.63 and 1835.85 (Fig. S2B). The unique m/z peak of 1129.63 reflects the myristoylation of m/z peak 919.43 and thus a mass shift of ∼210 Da corresponding to the formation of the myristoylated peptide. Our results demonstrate that N-terminal peptide region of HK1S is a valid substrate of NMT *in vitro* despite lacking Ser^6^/Thr^6^.

We further sought to establish whether the full-length HK1S is myristoylated at Gly^2^ during its ribosomal synthesis. We generated a C-terminal DDK-tagged version of HK1S (HK1S^DDK^) and a mutant for the Gly^2^ myristoylation site, HK1S^(G2A)DDK^. The full-length proteins were synthesized in a cell-free system employing rabbit reticulocyte lysates, an established system for co-translational N-myristoylation (Takamitsu et al., 2014). We employed TNT *in vitro* coupled transcription-translational system in conjugation with metabolic incorporation of the bio-orthogonal myristic acid analogue. Subsequently fluorescent labeling by sensitive ‘click-chemistry’ was employed for the identification of the myristoylation status of the full–length HK1S proteins (HK1S^DDK^ and HK1S^(G2A)DDK^). The cell-free transcription-translation was performed in the presence of myristic acid azide (50 μM) and examined for the synthesis of the full-length polypeptide. A single polypeptide band corresponding to the expected molecular size of ∼106 kDa of HK1S was observed in both HK1S^DDK^ and HK1S^(G2A)DDK^ (Fig. 2B, left panel). Following verification of the synthesis of full-length HK1S, to probe for the incorporation of azide-myristate, proteins captured on anti-DDK beads were subjected to copper-free ‘click-chemistry’ employing the strain promoted azide-alkyne cycloadditions with Alexa Fluor 488 DIBO alkyne. The fluorescence signal generated from the azide-alkyne conjugate could be detected in the labeled translation products from HK1S^DDK^ but not the HK1S^(G2A)DDK^ (Fig. 2B, right panel). The G2A mutant proteins abrogated the incorporation of the azide-myristate despite their successful synthesis, thus providing clear evidence that the HK1S protein is myristoylated in a co-translational fashion and that the myristoylation is mediated by the *Gly^2^* residue.

Travis et al. have reported that in the mouse HK1S (mHK1S), mutation of the (bold faced) residues in the motif ^15^**PK**I**R**PP**LTE**^23^ within the GCS region abolishes the membranous compartmentalization (Travis et al., 1999). We therefore extended our sequence conservation analysis to all of the GCS HK1 sequences available in the NCBI and Uniprot database (Pruitt et al., 2007; The UniProt Consortium, 2010). The alignment of complete GCS domain reflects very high polyspecificity within the aforesaid motif (Fig. S3A). A quantitative description of the amino acid variability (Shannon entropy) within the GCS domain reflects high randomness at positions encompassing the ^15^**PK**I**R**PP**LTE**^23^ motif of the mHK1S (Fig. 2C) and thus underscores any conserved functional role.

### Concomitant myristoylation and palmitoylation of HK1S regulate its localization to cell membrane

The binding energy provided by the myristoyl chain (*K*_d_ ∼10^−4^ M) is not sufficient to allow for the permanent association with membranes (Peitzsch and McLaughlin, 1993). Myristoylated proteins therefore have other membrane interaction motifs, which is most commonly a polybasic clustered region or S-acylation on a proximal Cys residue (Maurer-Stroh et al., 2004; Resh, 2006). The entropic analysis reflects that sequences proximal to the N-terminus, containing the N-myristoylation site, were significantly stabilized against randomness in comparison to the complete GCS domain (Fig. 2C). An absolute entropic stabilization at positions corresponding to the Cys^5^ residue is also observed (Fig. 2C, Fig. S3A). The Cys^5^ residue distal to the myristoylation site Gly^2^ is characteristically conserved within the consensus and could act as a putative site for palmitoylation as observed in numerous other proteins (Resh, 1999). We speculated that the specific atypical compartmentalization of HK1S operates by the dual-acylation signal in this region and the stable membrane targeting takes place through a direct liaison between Gly^2^ myristoylation with Cys^5^ palmitoylation. We also noticed that in addition to the sites for the myristoylation and palmitoylation, the positions adjacent to these residues (i.e. Gln^3^ and Gln^6^) are also stabilized against randomness (Fig. 2C, Fig. S3A). We therefore further examined whether the acylation sites, Gly^2^ and Cys^5^, in the motif ‘MGQICQ’ and the conserved Gln^3^ and Gln^6^ operate as a requirement for the atypical localizations of HK1S to PMP region.

**Fig. 3.**
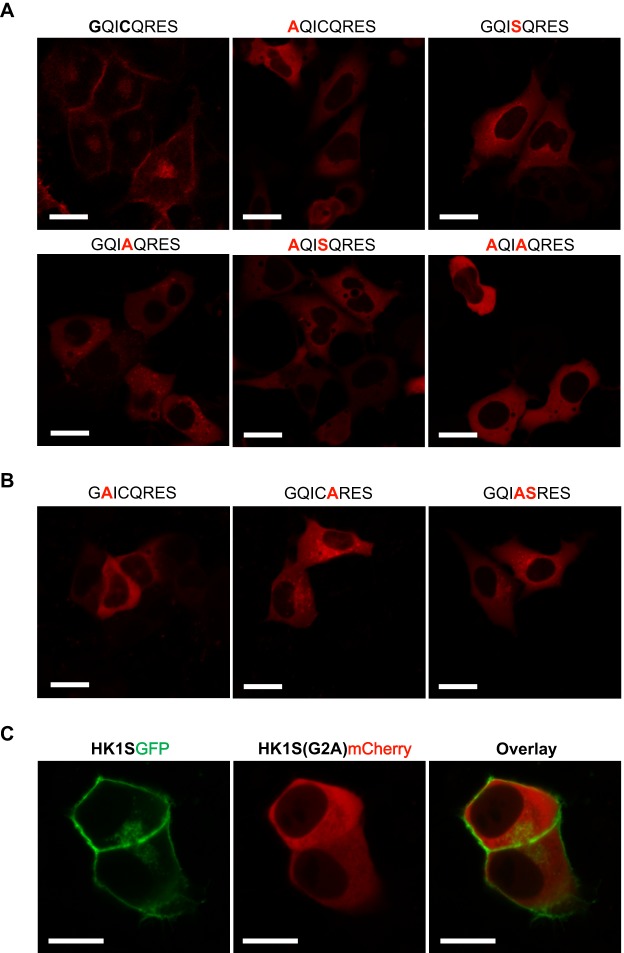
**Dual-acylation signal in GCS domain is critical for the distinct localization of HK1S.** (A) Live-cell confocal imaging of HEK293 cells transfected with HK1S^mCherry^ and the mutant proteins (mutations at the dual-acylation sites in the N-terminal region). The site-specific mutations at the sites of acylation Gly^2^ or Cys^5^ within the octapeptide region ^2^GQICQRES^9^ are indicated on the top of the panel (bold faced, red). (B) Effects on the localization pattern by mutations of the conserved sites Gln^3^ or Gln^6^. The mutations introduced are indicated on the top of the panel (bold faced, red) within the octapeptide region ^2^GQICQRES^9^. (C) The comparative loss of PMP localization depicted by a co-localization assay employing HK1S^GFP^ and HK1S^(G2A)mCherry^. Fluorescence was observed 24 h after transfection using Zeiss LSM 700. Scale bars=20 μm.

To verify our hypothesis, we engineered HK1S^mCherry^ mutants at the sites of acylation, Gly^2^ and Cys^5^, in which the mutations were introduced individually or in concert. To demonstrate the role of myristoylation and/or palmitoylation in HK1S atypical compartmentalization, HEK293 cells were transfected with wild type HK1S^mCherry^ and the mutants G2A, C5S, C5A, G2A/C5S and G2A/C5A, and examined by live–cell confocal microscopy (Fig. 3A). We observed that the wild type HK1S^mCherry^ is enriched in the PMP regions of the cell and to a lesser extent in the defined regions of cytoplasm (Fig. 3A; top left panel). However, mutations at the site of HK1S abrogating the myristoylation (G2A) or palmitoylation (C5S or C5A), either in isolation or in tandem, exhibited a more homogeneous distribution pattern in the cytoplasm indicating a loss of the peripheral compartmentalization as depicted in Fig. 3A. A larger field of cells corresponding to each of the mutations is shown in Fig. S4. Myristoylation acts as an essential prelude to direct the duly acylated protein for subsequent palmitoylation (Galbiati et al., 1994). Therefore, a dual-acylated protein is expected to reflect one of the three possible acylation states: (a) both myristoylation and palmitoylation, (b) myristoylation only, and (c) no acylation. The loss of the myristoylation site, in isolation, (G2A mutant) restricts the protein from undergoing palmitoylation and thus reflects a cytoplasmic localization (Fig. 3A, top central panel). Mutations at the site Cys**^5^** (i.e. C5S, C5A) results in mono-acylation status (i.e. only myristoylation) and show a punctate pattern within the cytoplasmic distribution (Fig. 3A, top right panel and bottom left panel); reminiscent of localization within an internal membrane. The double mutants of Gly^2^ and Cys^5^ reflect diffused cytosolic expression (Fig. 3A, bottom central and right panel) as expected by the complete loss of acylation. The loss of plasma membrane enrichment by mutations of either of the acylation sites reflects that both modifications are critical for the atypical targeting of HK1S to PMP and that they act in concert to promote stable membrane association.

We further analyzed the conserved sites Gln^3^ and Gln^6^ and a similar loss of compartmentalization is reflected (Fig. 3B, left and central panel). The residue at position 3 (i.e. Gln^3^ in HK1S) is a facilitator of myristoylation in absence of Ser^6^/Thr^6^ (Utsumi et al., 2001). Therefore, the mutation of Gln^3^ is expected to block myristoylation (and consequently palmitoylation). Accordingly, the Q3A mutant (Fig. 3B, left panel) displays cytoplasmic distribution similar to that of the mutant G2A (Fig. 3A, top central panel). Mutation of Gln^6^, Q6A, (Fig. 3B, central panel) appears alike to be of Cys^5^ mutants (C5S and C5A, Fig. 3A), suggesting a specific ablation of the palmitoyl incorporation with myristoylation being intact. We also introduced a Q6S mutation in the C5A mutant to correspond with the consensus myristoylation motif MGXXXS/T; however, the mutant protein failed to retain the compartmentalization (Fig. 3B, right panel) reflected by the wild-type protein (i.e. HK1S^mCherry^) (Fig. 3A, top left panel).

The relative enrichment of individual amino acids within the GCS motif (Fig. S3B) and the loss of PMP compartmentalization by mutations of either Gly^2^, Cys^5^, Gln^3^ or Gln^6^ (Fig. 3A and B) thus reflects that the consensus motif ‘MGQXCQ’ (Fig. S3B) in N-terminal of the GCS domain of HK1S is critical for its atypical localization to the PMP. Moreover, since myristoylation is required to direct palmitoylation, the myristoyl recipient Gly^2^ appears to act as the prime regulator to direct the atypical localization of HK1S. A comparative illustration of the complete loss of the compartmentalization by abrogating only the myristoylation site is shown by the co-localization of HK1S^GFP^ and HK1S^(G2A)mCherry^ (Fig. 3C).

## DISCUSSION

Life perpetuates by the fusion of sperm with the ova, for which spermatozoa are required to leave one organism, navigate long distances, and deliver their paternal DNA into a mature egg. For successful navigation and delivery, mammalian sperm, which is a highly specialized cell, has elaborate cytoskeletal structures in the tail for motility regulation. The mammalian sperm flagellum comprises >90% of the total length and motility is generated by the overall planar stroke, bend, and rotation of the flagellar tail. It is perceived to be modulated by outer dense fibers (ODFs) and the FS that separate the axoneme from the plasma membrane (Eddy et al., 2003). A substantial amount of ATP is required to support the coordinated movement of the central axoneme and surrounding flagellar structures. Furthermore,the ‘solid-state’ arrangement of glycolytic enzymes on the FS of sperm could produce ATP locally where it is needed to power flagellar motility and the regulators of that motility (Mukai et al., 2009). The oxidative phosphorylation is more efficient than glycolysis for ATP production; however, it is the glycolytic ATP that powers sperm along the entire length of the flagellum to support motility. The disruption of the FS-localized glycolytic enzymes consequently exhibit defects in sperm motility that therefore have sluggish movement resulting in male infertility (Danshina et al., 2010; Miki et al., 2004; Nakamura et al., 2013; Narisawa et al., 2002; Odet et al., 2013). The compartmentalized localization of HK1S is thus crucial to the sperm glycolytic pathway to ensnare the glucose in the ATP generation cycle. A compartmentalized arrangement of glycolytic enzymes specific to membrane-bound organelles is also observed in the protozoan parasite *Trypanosoma brucei* that increases the rates of ATP production 50 times in this organism to that of mammalian cells (Menard et al., 2014). The enhanced production of ATP locally by the compartmentalized FS bound enzymes, where it is needed to power the flagellar motility, would thus facilitate the biological roles of the sperm. As in mammalian sperm flagellum, an analogous layout is observed in the green algae *Chlamydomonas reinhardtii* where multiple glycolytic enzymes are targeted to the flagellar compartment and ATP is supplied along the length of the flagellum through glycolysis (Mitchell et al., 2005).

Our findings show that the HK1S association with the peripheral membranous region is an integral feature of the HK1S protein and does not require any anchor proteins localized to FS in sperm. We further show that HK1S is a novel myristoylated protein. The HK1S variant has been known for many years, but has not been previously predicted or suggested to be myristoylated. This is attributed to the fact that the N-terminal sequence of HK1S lacks a Ser^6^/Thr^6^; however, the presence of Ser^6^/Thr^6^ is not a strict requirement (Maurer-Stroh et al., 2002b). For instance, the catalytic subunit of mammalian cyclic AMP (cAMP)-dependent protein kinase, the first protein shown to be N-myristoylated, lacks the conservation of Ser^6^/Thr^6^ (Carr et al., 1982). In the absence of Ser^6^/Thr^6^, the presence of Gln^3^/Asn^3^ acts as a facilitator of myristoylation (Utsumi et al., 2001). This signature, present in the cAMP-dependent protein kinase (Carr et al., 1982), is also present in the HK1S acylation motif ‘MGQICQ’. Accordingly, in the presence of Asn^3^, incorporations of Ser^6^ in lieu of Ala^6^ to the peptide substrate ^2^GNAAAARR^9^ of cAMP-dependent protein kinase is known to affect the susceptibility to N-myristoylation (Maurer-Stroh et al., 2002b). This strict requirement of Gln^3^/Asn^3^ in the absence of Ser^6^/Thr^6^ is concomitantly conserved along with Gly^2^ in the N-terminal sequences of HK1S across the mammalian species (Fig. 3A, Fig. S3A). The absolute entropic stabilization of ‘MGQ’ (Fig. 3C) in the absence of Ser^6^ supports our notion that N-myristoylation is a conserved myristoylation process in the GCS region across the diverse mammalian HK1S. Nonetheless, this isoform of hexokinase has also remained unidentified in the recently reported global analysis of human proteome myristoylation (Thinon et al., 2014). It is of importance to note that the functional diversification of proteins increases by an alternative inclusion of tissue-specific protein-coding exons most notably in the testis and brain (Kimura et al., 2006). The global analysis of ‘myristoylome’ has focused on the normal and apoptotic states of a particular cell type (Thinon et al., 2014). The HK1S protein is specific to sperm and thus may have escaped the global myristoylome profiling. We therefore hypothesize that the full-spectrum of myristoylated human proteome is still beyond the horizon in diverse tissue types. This is exemplified by the myristoylation of HK1S, the myristoylation motif of which is the product of alternate gene transcription by sperm specific-alternative promoter of HK1, in a highly tissue-specific manner. Our findings conclusively show that the atypical compartmentalization of HK1S is regulated by novel myristoylation of the protein and operates through the myristoyl-palmitoyl switch mechanism (Resh, 1999). This is conferred on the sperm-specific novel N-terminal motif generated by the alternative splicing of the somatic isoform HK1. In summary, we show that the tissue-specific inclusion of a novel N-terminal segment that contains a dual-acylation motif is essential for atypical localization HK1S and thus essential for the ‘rewiring’ of the glycolytic network essential for sperm motility.

## MATERIALS AND METHODS

### Materials

Restriction endonucleases were obtained from MBI-Fermentas, *Pfu* DNA polymerase was from Stratagene and DNA ligases were from New England Biolabs (NEB). *E. coli* strain NEB 5-α was used for cloning of cDNAs and amplification of plasmids. Oligonucleotides used in the generation of expression constructs, MitoTracker Green FM, myristic acid azide (12-azidododecanoic acid; *Az*Myr), Alexa Fluor 488 DIBO Alkyne, cell culture reagents, and Lipofectamine LTX (with PLUS™ reagent) were obtained from Invitrogen. Reagents for SDS-PAGE and western blotting were from Bio-Rad. The peptide substrate corresponding to the N-terminal of human HK1S (^2^GQICORES^9^-CONH_2_) was custom synthesized and obtained from the Institute for Biomolecular Design (University of Alberta). Myristoyl-CoA (lithium salt) was purchased from Sigma-Aldrich. PCMV6-Entry vector, anti-DDK monoclonal antibodies and anti-DDK Agarose beads were purchased from Origene. Anti-mouse antibodies linked to infrared-dyes (IRDye800CW) were from LI-COR Biosciences. Live cell imaging 8 chamber μ-slides were obtained from Ibidi. Human embryonic kidney cells (HEK293) cells originally from the American Type Culture Collection were obtained from shared laboratory resources (Cancer Cluster, University of Saskatchewan). All other reagents were from Sigma-Aldrich unless otherwise indicated.

### Generation of plasmid constructs

The coding sequences for the green and red fluorescent (GFP and mCherry, respectively) proteins were amplified from the plasmids pEGFP-C1 (Clontech) and pmCherry-C1 (Clontech) using the primers 5′-CTACCGGATATCCTCGAGGTGAGCAAGGGCGAGGAG-3′ (forward) and 5′-GCTCGAGATGGCCGGCCTTACTTGTACAGCTCGTCCATG-3′ (reverse) against the homologous amino acid at N- and C-terminal regions (VSKGEE and MDELYK, respectively), containing *Xho*I and *Fse*I restriction sites, respectively (underlined). The fluorescent proteins were cloned in the mammalian expression construct pCMV6-Entry (Origene) resulting in template plasmids pCMV6^GFP^ and pCMV6^mCherry^. To obtain the coding sequence for human HK1, total RNA was extracted from HEK293 cells using the Trizol reagent (Invitrogen) followed by reverse transcription with 1 μg of aliquots of the transcribed RNA using SuperScript first-strand synthesis system for RT-PCR (Invitrogen) according to the manufacturer's instructions. Partial cDNA transcript of HK1 corresponding to amino acids 175-917 of HK1N (CCDS ID: CCDS7292.1) was obtained using *Pfu* DNA polymerase employing forward (5′-GATTTAAAGCTAGCGGAGTGGAAGGAGCAG-3′) and reverse (5′-AGCGGCCGTTACTCGAGGCTGCTTGCCTCTGTG-3′) primers containing *Nhe*I and *Xho*I restriction sites, respectively (underlined). The *Nhe*I site in forward primer is a conservative substitution of Asp-Ser codons in the amino acid region ^175^FKASGVEGA^183^ of human HK1 cDNA (CCDS ID: CCDS7292.1). The PCR amplified product was digested with *Nhe*I*-Xho*I enzyme pair and ligated to pCMV6^GFP^ resulting in plasmid pCMV6^ΔN-HK1GFP^. The DNA fragments corresponding to the N-terminal portions of the HK1N (amino acid 1-184) and HK1S (amino acids 1-188) (CCDS ID: CCDS7292.1 and CCDS7289.1, respectively) were obtained as synthetic gene fragments (Table S1) and contained N-terminal extensions corresponding pCMV6-Entry plasmid (Origene). The portions were PCR amplified using the primers 5′-GAATTCGTCGACTGGATCCGGTACCGAGGAGATCTGC-3′ (forward) and 5′-TCCACTCCGCTAGCTTTAAATCGCTTTGTCCAGGTG-3′ (reverse) containing *Kpn*I and *Nhe*I restriction sites (underlined) and cloned to pCMV6^ΔNHK1GFP^ resulting in GFP fusion constructs of HK1N and HK1S, pCMV6^HK1NGFP^ and pCMV6^HK1SGFP^, respectively. For the generation of pCMV6^HK1SmCherry^, the full-length coding sequence of HK1S was spliced from pCMV6^HK1SGFP^ using the sites *Kpn*I and *Xho*I and cloned to pCMV6^mCherry^.

Plasmid encoding for the C-terminal DDK tagged HK1S (pCMV6^HK1SDDK^) was generated by the cloning of the *Kpn*I*-Xho*I fragment from pCMV6^HK1SGFP^ to the *Kpn*I*-Xho*I digested pCMV6-Entry vector (Origene). For introducing site-specific mutations in the N-terminal region of the GCS domain of HK1S, forward primers with designated nucleotide changes coding for the desired mutations (Table S2) and reverse primer 5′-TCCACTCCGCTAGCTTTAAATCGCTTTGTCCAGGTG-3′ was used to amplify the N-terminal portion of HK1S using pCMV6^HK1SDDK^ as template and cloned to *Kpn*I*-Nhe*I digested pCMV6^HK1SDDK^ or pCMV6^HK1SmCherry^ constructs as designated. All plasmid constructs were subjected to automated DNA sequencing to verify that no undesired mutations were introduced.

### *In vitro* myristoylation and proteomic identification of myristoylated HK1S peptide

N-terminal peptide, ^2^GQICQRES^9^-CONH_2_, of human HK1S was subjected to the previously described *in vitro* myristoylation assay with minor modifications (Kumar and Sharma, 2014). In brief, the *in vitro* myristoylation reaction was carried essentially as before; however, employing the catalytic domain of NMT1 lacking the N-terminal inhibitory region (Kumar and Sharma, 2015). The reaction mixture lacking the myristoyl-CoA served as control. Aliquots of the reaction products (1 μl) were spotted directly to the MALDI matrix and analyzed by mass spectrometry as described earlier (Kumar and Sharma, 2014).

### Cell-free synthesis, metabolic labeling and ‘click-chemistry’ enabled detection of HK1S myristoylation

Cell-free synthesis of full length HK1S was performed by using the TNT quick-coupled transcription/translation systems (Promega) according to the manufacturer's instructions. Briefly, HK1S^DDK^ and HK1S^(G2A)DDK^ were transcribed and translated in a TNT quick master mix containing rabbit reticulocyte lysate. Bio-orthogonal metabolic labeling was carried during the synthesis by performing the reactions (30°C, 90 min) in the presence of 50 μM *Az*Myr. The reaction mixture was diluted five-fold with PBS (pH 7.0) and the translated polypeptides were captured on pre-equilibrated anti-DDK agarose beads. After the immunoprecipitation, aliquots of immunocomplexes were separated by SDS-PAGE (12%) and then electroblotted onto a nitrocellulose membrane. The synthesis of full-length proteins was detected using anti- DDK monoclonal antibodies (1:1500 dilution) and probed with appropriate secondary antibodies as described earlier (Kumar and Sharma, 2014).

Following verification of the synthesis, immunocomplexes were washed extensively with PBS and processed for labeling with Alexa Fluor 488 DIBO Alkyne using cyclooctyne azide-alkyne Click-iT Reaction (Invitrogen) according to the manufacturer's instructions. The click reactions were performed on the proteins bound to anti-DDK beads. The fluorescently labeled reaction products were resolved by 12% SDS-PAGE and transferred to nitrocellulose to enhance the signal intensity. The signals of Alexa Fluor 488 were observed on Molecular Imager *FX* (Bio-Rad) utilizing the FITC fluorescence channel.

### Cell culture, plasmid transfections and live-cell confocal microscopy

HEK293 cells were routinely cultured in Dulbecco's modified Eagle's medium (DMEM) supplemented with 10% fetal bovine serum (FBS), 100 μg/ml streptomycin, and 100 units/ml penicillin. The cells were grown at 37°C in a humidified atmosphere with 5% CO_2_. For confocal analysis, 24 h prior to transfection, HEK 293 cells were seeded at a density of ∼7.5×10^4^ cells/chamber in the 8 chamber μ-slides (Ibidi). The cells were transiently transfected with HK1N or HK1S (or both) expression constructs (fused to either GFP or mCherry) using Lipofectamine LTX (with PLUS™ reagent) according to the manufacturer's instructions. Twenty-four hour post transfection, cells were imaged directly as live cells at 37°C in a humidified 5% CO_2_ atmosphere using a Zeiss LSM700 confocal microscope. Nuclei staining were performed using the live-cell permeable nuclear dye [Hoechst 33258; 10 μM dye (final concentration) incubated for 15 min at 37°C]. All image adjustments (gain, pinhole size, or background) were optimized using the LSM700 Meta software; pictures were exported as 8-bit color images and processed with Adobe Photoshop CS3.

### Amino acid conservation and Shannon divergence in the GCS domain

For sequence conservation and Shannon entropy analysis, thirteen orthologous sequences corresponding to the GCS domain of HK1S were retrieved from Uniprot and NCBI databases (Pruitt et al., 2007; The UniProt Consortium, 2010). Multiple sequence alignments were performed using PRALINE with an optimized heuristic methodology using the PSI-BLAST pre-profile processing (homology-extended alignment) with a gap-opening penalty of 12 and an extension penalty of 1. The alignment was assessed based on the amino acid conservation index; scored with 0 for the least conserved, to 10 for the most conserved, alignment position. For the analysis of the significance of positional amino acid restrictions, the multiple sequence alignment data was analyzed using the Shannon information entropy measure (*Hx*) (Capra and Singh, 2007; Strait and Dewey, 1996). For a multiple protein sequence alignment, the (*Hx*) for every position (*x*) is given by the relation,

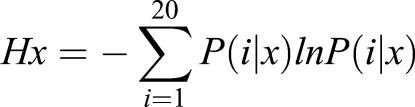
where, *P*(*i*|*x*) is the conditional probability of residue type (*i*), occurring at alignment position (*x*). The Shannon index (*Hx*) ranges from 0 (only one residue in present at that position) to 2.996 (all 20 residues are equally represented in that position).

The sequence logo was generated with the WebLogo 3 application (http://weblogo.berkeley.edu/).
